# Land consumption and income in Ecuador: A case of an inverted environmental Kuznets curve^☆^

**DOI:** 10.1016/j.ecolind.2019.105699

**Published:** 2020-01

**Authors:** Nicola Pontarollo, Rodrigo Mendieta Muñoz

**Affiliations:** a European Commission, Joint Research Centre (JRC), Ispra, Italy; b Universidad de Cuenca, Cuenca, Ecuador

**Keywords:** Environmental Kuznets curve, Economic development, Land use, Ecuador, Spatial econometrics

## Abstract

The ratio of building permits to population is a key indicator to evaluate land consumption. However, few researchers focus on land consumption and its environmental spillovers, for developing countries. The aim of our study, using a Bayesian comparison approach applied to a spatial panel, is to analyse the existence of an inverted U-shaped curve relationship between land consumption and economic development, namely the environmental Kuznets curve, with data that ranges from 2007 to 2015 for 221 cantons in Ecuador. The Bayesian comparison approach allows us to identify: i) the spatial weight matrix that best fits the data, and ii) the best spatial model according to the type of spatial spillovers (local or global). These are both of extreme interest because a knowledge of the extent to which the spatial spillovers spread over space, and their functional form, supports the planning of effective land use policies. The results do not support the inverted U-shaped hypothesis of the Kuznets curve. By contrast, the curvature is convex, which means higher levels of land consumption for higher levels of wealth. Spatial spillovers spread to a limited extent, highlighting an imitation game among agents, both institutions and private agents, in the neighbour locations. Policy implications go from the strengthening of the institutional framework and local tax management, to the urban regeneration to limit real estate speculation. All these interventions should be coordinated among neighbours to avoid freeriding behaviours.

## Highlights

• We use the spatial panel regression model to estimate the land Kuznets curve.
• We examine spatial auto-correlations in the building permits in Ecuador.
• A Bayesian selection method allows selecting the right spatial model and weight matrix.
• Economic development has a U-shape relationship with land use.
• Spatial spillover effects have a limited extent and affect only closest neighbours.

## 1 Introduction

Between 1990 and 2015, population in urban centres increased by 50% in Latin America, against 44% in the world (Pesaresi et al. 2016). According to the UN-HABITAT (2012), 84% of the South American population lives in urban areas, more than in North America (82%) and Europe (73%). By 2050, it is expected that 90% of the South American population will be urban inhabitants (UN-HABITAT, 2012). Inostroza et al (2013), analysing a number of Latin American cities, find that these cities tend to concentrate most of their built-up area in the core, but that, together with the economic development, a segmentation process, which leads to the separation of core from fringe areas is occurring.

According to IPBES (2018), such urban expansion contributes to the habitat degradation due to land conversion. The declining biodiversity and ecosystem conditions, in turn, have an effect on reducing nature’s contributions to peoplés quality of life in many parts of the Americas (IPBES, 2018).

Soil is a non-renewable resource, due to its slow regeneration process (Pimentel et al., 2010, Gardi et al., 2015). This means that spatial planning has to make the best use of land and soils by regulating the access and limiting overuse for the common welfare. In this regard, economic development has been proved to have a close correlation with land use changes and its degradation (Jedwaba and Vollrathb, 2015, Bimonte and Stabile, 2017a, Bimonte and Stabile, 2017b).

In our study, we test the existence of an inverted U-shaped curve relationship between land consumption and economic development, namely the environmental Kuznets curve (EKC), in Ecuador. We focus on Ecuador because, as in many other Latin American countries, Ecuador has experienced rapid economic growth and rising urbanization (Angel, 2008, UN-HABITAT, 2012). According to the EKC, economies have a concave relation with environmental degradation, meaning that the closer they are to the turning point at which economic development causes an environmental improvement, the less economic growth deteriorates the environment.

Ecuador can be considered an interesting case study because it does not only share some common features with other countries in the region (e.g. rapid economic growth due to a raw materials super cycle), but has some other characteristics worth mentioning.

These refer in particular to the urbanization process, which is above the Latin American average for the period 1995–2015 (UN-HABITAT, 2016), and to the fact that “the new Constitution formally recognizes natural environments as ‘political subjects’, with local people acting as official agents. This reverses humankind’s conventional relationship to nature, not just redistributing power and responsibilities to urban residents but also, just as importantly, ushering current and future generations into a newly found, global history of nature” (UN-HABITAT, 2016: 84). The mentioned political framework is set out in Section III, Chapter I of the Organic Code of Territorial Organization, Autonomy and Decentralization (COOTAD), and is intended to devolve to local governments the tasks of stemming excessive land use (Art.55).

In our study, we rely on data that spans the period from 2007 to 2015 for 221 cantons.^1^ The indicator of land use, in line with Bimonte and Stabile, 2017a, Bimonte and Stabile, 2017b, is the ratio of building permits to population. This indicator, to our knowledge, has not been used for Latin American countries and at this level of disaggregation, allowing us to analyse the space–time characterization of land use in relation to economic development, and the possible environmental implications. Compared with previous studies, e.g., Wang et al., 2013, Liu and Guo, 2015, Hao et al., 2018, Zhou and Wang, 2018, we innovatively contribute to the literature applying a Bayesian comparison approach applied to a spatial panel model. The first advantage of this technique is that a spatial panel model, based on a spatial weight matrix, which models the spatial connectivity among administrative areas, allows us to identify spatial interaction and feedback effects (namely, spillovers) between locations. Spillovers exist when phenomena occurring in places close to each other are not independent, i.e. when a shock on a variable in a determined a location affects the variable in that location, and also the neighbour areas. The second and key advantage of our model is that the Bayesian comparison approach allows us to identify: i) the spatial weight matrix that best fits the data, and ii) the best typology of spatial model. These two information are of fundamental importance because, while other mentioned studies rely on a single exogenously predetermined spatial weight matrix and test few functional forms of spatial models, we identify the best spatial weight matrix and model by checking from a wide set of different options and combinations of the two. The importance of the spatial weight matrix is stressed by Florax and Rey, 1995, Franzese and Hays, 2007 who prove that if it is misspecified, it has a non-negligible effect on the coefficients of the model. On the other hand, the different models accommodate spatial dependence in different forms, which lead to very different policy implications (see paragraph 3.2). Our approach, that allows us to model with a higher degree of precision and confidence the extent to which the spatial spillovers spread over space, and their functional form, may let policy makers to plan more effective land use policies.

Spatial spillovers, or interaction, indeed, can play a strong role into explaining land use and its evolution. Anas et al., 1998, Krugman, 1996 show that the evolution of cities is affected by spillovers that generate varying sizes and locations of clusters of households, firms, and infrastructure. Proximity to other firms and workers generates diffusion of knowledge (Duranton and Puga, 2004) whose cost of transmission increases with distance (Audretsch and Feldman, 1996). As the knowledge transfer mechanisms are only effective when the distance to stakeholders (customers, suppliers, competitors etc.) is small (Von Hippel, 1988, Von Hippel, 1994), firms tend to cluster (Feldman, 1999). Possible positive environmental effects of this process are shown by Hájek and Stejskal, 2018, Aldieri and Vinci, 2017 using microdata on companies. Indeed, the former show that knowledge spillovers may generate innovation through internal collaborations and cooperation with private and public actors that can contribute to generate sustainable eco-innovations. The latter point that specialization in environmental technology generate positive environmental spillovers on productivity and environmental performance. Antonioli et al. (2016), on the other hand, demonstrate local spillovers of a sector/geographical nature are crucial for the diffusion of environmental innovations and for economic performances of firms. Excessive spatial concentration, however, may cause congestion externalities making households and firms to disperse (Tabuchi, 1998), generating a range of patterns from compacting to sprawling (Page, 1998). The latter has deep environmental consequences which range, for instance, from fragmentation (Razin and Rosentraub, 2000) and habitat loss (Scolozzi and Geneletti, 2012) to an increasing air pollution (Liu et al, 2018) and electricity consumption (Lasarte Navamuela et al, 2018).

The concept of spatial spillovers can be applied also to land use policymaking, and it regards the strategic interaction among local planning officials (Brueckner, 1998, Schone et al., 2013, Shipan and Volden, 2008).

Worldwide, few studies have related land use to economic development (Liu and Guo, 2015; and Bimonte and Stabile, 2017a, Bimonte and Stabile, 2017b) and, to the best of our knowledge, this relationship has not been explored for Latin America and the Caribbean (LAC), and for Ecuador in particular.

This paper, therefore, contributes to the literature on the EKC both by applying the spatial econometric approach to a developing country, and by focusing on a still poorly explored variable, land use, which has deep environmental and social implications.

Studies on LAC have provided mixed results with respect to the existence of the environmental Kuznets curve. Authors like Pao and Tsai, 2011, Robalino-López et al., 2014a, Al-Mulali et al., 2015, and Hanif (2017), among the latest, confirm the hypothesis of the inverted U-shaped curve, using different methodologies, time periods and environmental indicators generally related to pollutants. Studies such as Robalino-López et al., 2015, Zambrano-Monserrate et al., 2017 for pollutants, and Pablo-Romero and De Jesús (2016) for energy consumption, however, do not support the existence of an environmental Kuznets curve relationship for the countries of this region. Other authors examining the EKC in a wider selection of countries also find mixed evidence for its existence. Özokcu and Özdemir (2017), analysing 26 OECD countries with high income and 52 emerging countries demonstrate that the first group has an inverted N-shaped curve, while the second group has an N-shaped relationship between CO_2_ emissions and GDP per capita. On the other hand, Bilgili et al., 2016, Azam and Khan, 2016 find a U-shaped relationship for some countries and an inverted U-shape for other countries, while performing single country analyses.

Some studies have examined the Ecuadorian reality. Zambrano-Monserrate et al. (2016), using an autoregressive distributed lag bounds testing approach, find evidence of a long-run environmental Kuznets curve (EKC) for Ecuador from 1971 to 2011 for economic growth and for energy on carbon dioxide emissions (CO_2_). Nwani (2017), instead, finds a unidirectional causality that flows from CO_2_ emissions to economic growth and confirms the positive and statistically significant long-run effect of energy consumption on CO_2_ emissions. Robalino-López et al. (2014a), using a variation of the Kaya identity, find that it is possible to control the CO_2_ emissions if GDP growth is combined with an increase in the use of renewable energy. Based on the previous study, Robalino-López et al. (2014b), use a cointegration approach to prove that the EKC does not hold in Ecuador, while Al-Mulali et al. (2015) show the existence of an EKC between CO_2_ and GDP in Ecuador and other Latin American countries through the FMOLS and VECM Granger causality techniques. Finally, Jin et al. (2016), employing annual data covering the period 1965–2011, and using unit roots, co-integration and VECM Granger causality approaches, find unidirectional causality running from economic growth to oil consumption. Worth mentioning is that none of these studies account for spatial spillovers.

This paper is structured in five sections. The second section deals with land use in Ecuador, the third section describes the empirical strategy and the estimation technique of the model, while in fourth section we illustrate the empirical results of our analysis. In the last part, finally, we presented the discussions and conclusions.

## 2 Land use in Ecuador

Land use is regulated by the National Territorial Strategy (NTS), which comprises short, medium and long-term criteria, guidelines and actions on the physical ordering of the country’s territory and its natural resources. It contains rules for the establishment of infrastructure and spatial development, the promotion of economic activities, as well as the protection and conservation of the natural and cultural heritage. The Ministry of Urban Development and Housing sets the main priorities, which include the promotion of inclusive polycentric territorial development and safe and adequate housing, establishing the policy guidelines for cities. The responsibility of the implementation of the NTS is shared by three administrative levels: zones, provinces and cantons. The latter have political autonomy, collection and spending capacity, and regulate the use and occupation of urban and rural land.

One consequence of the NTS is that its application requires very fine data on land use, and the collection of this data has improved notably in the last decade (National Secretary of Planning and Development, SENPLADES, 2017). For this paper, we rely on building permits, which are provided by the National Statistical Institute. Each building permit contains a number of characteristics that allowed us to select only new building construction, excluding restructuring and other types of interventions.

**Fig. 1:**
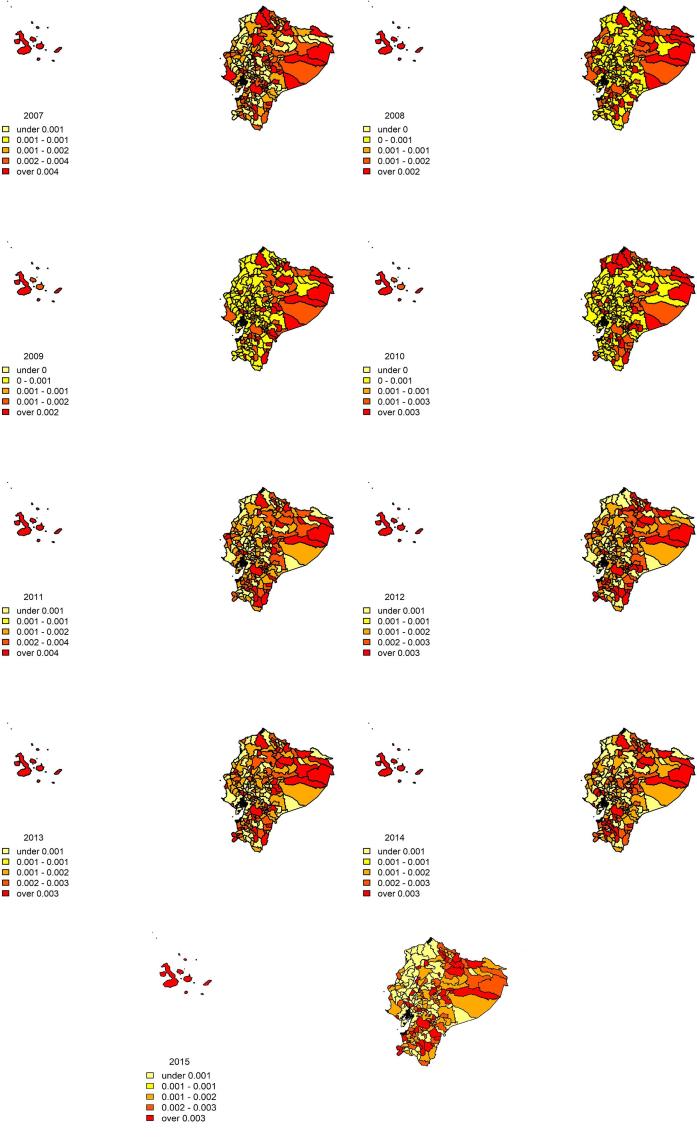
land use in Ecuador (number of building permits according to the population).

The distribution of building permits according to the population is shown in Fig. 1. We observe a generalized increase between 2011 and 2014, with the highest share of permits in the northern, southern and eastern parts of the country. The lowest, meanwhile, is along the coast.

The statistical significance of the spatial pattern of land use described above can be determined through the well-known Moran’s I,^2^ and this shows a significant positive spatial autocorrelation for all the considered years (i.e. areas with a similar distribution of building permits according to their population are likely to be located close to each other). In particular, there is a positive spatial autocorrelation equal to 0.17 in 2008, 0.13 in 2010 and 0.10 in 2012, while in the remaining years it ranges between 0.027 and 0.061. Besides this, it is interesting to observe that the Gini index^3^ performed on cantonal land use, which, in this context gives us a measure of the spatial inequality, has very high values, between 0.86 and 0.93.

The analysis of these two measures together tells us that in Ecuador land use tends to be spatially polarized by nature and significantly but limitedly clustered in space, which means that there are some circumscribed places (groups of cantons) where the share of new building permits are higher than in other areas of the country. This might find an explanation in Blanco et al.’s (2014: 7), who argue that, for LAC, “policy interventions have also contributed to spatial segregation by creating incentives for the construction of low-income housing on the outskirts of cities.” Jaitman (2015) adds that, generally, in the whole of Latin America low-density suburban areas are rising. In Ecuador, this phenomenon is resulting in agricultural lands being converted to low-density suburban areas, as is happening, for example, in Quito (Parés-Ramos et al., 2013).

## 3 Empirical strategy

### 3.1 Empirical model

The empirical model is a spatial extension of Bimonte and Stabile, 2017a, Bimonte and Stabile, 2017b. We select a Spatial Lag Panel Model that relates a variable in a certain location *i* to the same variable in neighbour locations, and to other covariates. The choice of the Spatial Lag Panel Model is made comparing it to a set of alternative spatial models, as specified below. The spatial Lag Panel Model is as follows:

(1) $$y_{i,t}=\rho W_{t}y_{t}+\psi log{\left(\frac{\mathrm{GVA}}{\mathrm{pop}}\right)}_{i,t}+\gamma {\left[\log{\left(\frac{GVA}{pop}\right)}_{i,t}\right]}^{2}+\theta log{\left(\frac{\mathrm{pop}}{\mathrm{area}}\right)}_{i,t}+\vartheta {\left(share\;mkt\;sector\right)}_{i,t}+\mu_{i}+\tau_{t}+\varepsilon$$

where *i* is the i^th^ canton of which there are *n*, and *t* the year of which there are *T*. $W_{t}$ is a squared spatio-temporal spatial weight matrix defied as the Kronecker product between the the identity matrix ***I****_T_* of size *T* and the spatial weight matrix **W** of size *n* × *n*. Formally, **W***_t_* = **I***_T_* ⊗ **W**; $\mu_{i}$ is the vector of spatial fixed effect (which embodies the unexplained time invariant characteristics); $\tau_{t}$ is the vector of a time-period fixed effect; $\varepsilon_{t}$ is the idiosyncratic error term. The term ***y****_t_* represents the log of the building permissions over population, *GVA/pop* the Gross Value Added per capita in 2007 constant price US dollas (USD) and *pop/area* and *share mkt sector* are two control variables representing population density and the share of GVA in the market sector (manufacturing and financial services over total GVA),^4^ respectively. The data for GVA are from the Central Bank of Ecuador, 2015, Central Bank of Ecuador., 2015 and building permission and population from the National Statistical Institute (INEC).

In line with the hypothesis of Kuznets (1955), the log of GVA per capita is included non-linearly. According to Kuznets’ theory, GVA per capita should be concave and significantly different from zero. This implies that, at a certain stage of development, land consumption starts diminishing (Skonhoft and Solem, 2001, Culas, 2007, Liu and Guo, 2015). In order to reduce misspecification problems, we control for other possible determinants of land use: population density and share of market sector. Population density is supposed to increase land consumption because more people require more housing; market sector, instead, might generate positive effects in the local economies, including inbound migration that can increase housing demand. We have not been able to include additional sectors because of the limited amount of data provided at cantonal level.

Equation (1) is a Spatial Lag Panel Model with spatial and time-period fixed effects (see, for instance, Elhorst, 2014). Since this cannot be estimated through an Ordinary Least Squares (OLS) method, it is here estimated with a maximum likelihood approach (ML) (Anselin, 1988). For a robustness check, finally, we follow Bouayad-Agha and Védrine (2010) estimating a spatial lag dynamic panel using a Generalized Method of Moments (GMM) approach.

### 3.2 Model and spatial weighting matrix selection strategy

Even though the selection of the Spatial Lag Panel Model as our baseline model is based on the idea that land use in a location is positively related to the use in neighbouring locations, we apply Bayesian estimation methods to solve model comparison problems formally (LeSage, 2014, LeSage, 2015). This allows us to compare models that account for different forms of spatial dependence, and based on various spatial weight matrices **W**.

According to Anselin (2002), the spatial dependence can take two different forms: nuisance and substantive. If we have nuisance, the error term is nonspherical (Anselin 1988), leading to inefficient but unbiased ordinary least squares (OLS) estimates. To deal with this issue, the use of the so-called Spatial Error Model allows to deal with spatial autocorrelation in the error term. When the spatial dependence has a substantive interpretation, the variable of interest at one location is jointly determined by its values at other locations. In this case we can have local or global spillovers (Anselin, 2003). Local spillovers occur when a change in an independent variable in a neighbour location affects other spatial units located in the immediate neighbours (but not beyond), i.e. only the areas that according to **W** are connected to each other. This is the case of Spatial Lag of X and Spatial Error Durbin Model. The models that include a spatial lag, or autoregressive, term, i.e. a weighted sum of the values of the dependent variable at the neighbour locations, produce global spillovers. This means that a change in an independent variable *X* of any spatial unit is transmitted to all other units, also if they are not connected according to **W** (the mechanism of transmission is explained in detail in the following paragraph). If substantive spatial dependence is not accounted for, the results are biased and inefficient.

Regarding the spatial weight matrix **W**, Anselin (2002: 259) affirms that there is no “correct” functional form for its specification. **W**, in fact, can be defined in many functional forms, based generally on contiguity and distance (Beck et al. 2006). Furthermore, even though the spatial weight matrix is important in determining the appropriate form of the spatial model, and regression results depend on it (Plümper and Neumayer, 2010), **W** is usually modelled exogenously and defined *a priori* (Wang et al., 2013, Liu and Guo, 2015, Hao et al., 2018, Zhou and Wang, 2018).

These considerations may generate uncertainty both in respect to the choice of the best spatial models and related spillover processes, and in respect to the choice of the spatial weight matrix.

To overcome these limitations, in the Bayesian model comparison, we consider the following models:

(2) $$\begin{array}{c} \mathrm{Spatial}\;\mathrm{Lag}\;\mathrm{Model}\;(\mathrm{SLM}):\\ y_{i,t}=\rho W_{t}y_{t}+\mu_{i}+\tau_{t}+\varepsilon \end{array}$$

(3) $$\begin{array}{c} Spatial\;Lag\;of\;X\;Model\;\left(SLX\right):\\ y_{i,t}=\delta W_{t}X_{t}+\mu_{i}+\tau_{t}+\varepsilon \end{array}$$

(4) $$\begin{array}{c} Spatial\;Durbin\;Model\;(SDM):\\ y_{i,t}=\rho W_{t}y_{t}+\delta W_{t}X_{t}+\mu_{i}+\tau_{t}+\varepsilon \end{array}$$

(5) $$\begin{array}{c} Spatial\;Error\;Model\;\left(SEM\right):\\ y_{i,t}=X+\mu_{i}+\tau_{t}+\eta \;with\;\eta =\lambda W_{t}\eta +\varepsilon \end{array}$$

(6) $$\begin{array}{c} Spatial\;Error\;Durbin\;Model\;\left(SDEM\right):\\ y_{i,t}=X_{t}+\delta W_{t}X_{t}+\mu_{i}+\tau_{t}+\eta \;with\;\eta =\lambda W_{t}\eta +\varepsilon \end{array}$$

where $X_{t}$ is the vector of dependent variables.

Regarding the spatial weight matrix **W**, we test four alternative contiguity schemes:

• k-Nearest Neighbours: $w_{i,j}=\left\{\begin{array}{c} 1\;\mathrm{if}\;\mathrm{canton}\;i\;∊\;\mathrm{to}\;\mathrm{the}\;\mathrm{set}\;\mathrm{of}\;\mathrm{the}\;k\;\mathrm{nearest}\;\mathrm{neighbours}\\ 0,\;\mathrm{otherwise} \end{array}\right)$
• Bisquare: $w_{i,j}=\left\{\begin{array}{c} {\left(1-\left({\mathrm{dist}}^{2}/{\mathrm{cutoff}}^{2}\right)\right)}^{2}\;\mathrm{if}\;dist\leq cutoff\\ 0,\;\mathrm{otherwise} \end{array}\right)$
• Gaussian: $w_{i,j}=\left\{\begin{array}{c} \exp\left(-\left(\frac{\mathrm{dist}}{\mathrm{cut}\mathrm{off}}\right)\right)\;\mathrm{if}\;dist\leq cutoff\\ 0,\;\mathrm{otherwise} \end{array}\right)$
• Weighted k-Nearest Neighbours: the spatial weight matrix is derived from a k-Nearest Neighbours, and each point is weighted according to the Bisquare scheme, where the cutoff for each row is set based on the k-Nearest Neighbour with the maximum distance.

Once the contiguity schemes have been estimated, the **W** matrices have to be normalized.^5^ The k-Nearest Neighbours weighting scheme is standardized following the normal row standardization. The latter, according to Elhorst (2014), has some shortcomings: the spatial weight matrix may become asymmetric and, perhaps more important, remote and central regions will end up having the same impact, i.e. independent of their relative location. Thus, in order to maintain the economic interpretation in terms of distance decay, for the last three weighting schemes we employ the method introduced by Kelejian and Prucha (2010), where the normalization is made by dividing **W** by its maximum eigenvalue**.**

The selection of both the spatial model and spatial weight matrix is based on a Bayesian model comparison for static panel models (LeSage, 2014, LeSage, 2015). For model M_k_, of which there are K, p(y|M_k_) is the marginal likelihood and p(M_k_) is the prior probability. According with LeSage (2014), the difference between the prior probabilities assigned to the models and the posterior model probabilities reflects Bayesian learning about the model specification conditional only on the sample data.

The marginal likelihood for model M_1_ obtained by integrating over *δ*_1_ is

(7) $$p\left(y|M_{1}\right)=\int p\left(y|\delta_{1},M_{1}\right)p\left(\delta_{1},M_{1}\right)d\delta_{1}=\int \left(likelihood\;x\;prior\right)d\delta_{1}$$

where we use non-informative prior distributions because we compare models with the same variables. Based on this criterion, the model and corresponding spatial weight matrix with the highest marginal likelihood are chosen.

### 3.3 Spatial effects

In the Spatial Lag Panel Model the impact of a change in a regressor on the dependent variable is a combination of the so-called direct and indirect effects mediated by neighbours’ influence (see Elhorst, 2010, Elhorst, 2014). Following LeSage and Page (2009), the *direct effects* are the effects of a marginal increase of a variable in a certain spatial unit *i* on the dependent variable of the unit itself (e.g., the GVA per employee in the same province *i*). In a Spatial Lag Model, this is actually the result of local effects plus feedback effects mediated by spatial spillovers.^6^ The *indirect effects* are the effects that the change of a variable in a certain unit *i* produces on the dependent variable of the other units.

In particular, taking the matrix of partial derivatives of the expected value of ***y***_t_ with respect to the explanatory variables, we have:

(8) $$\frac{\partial y}{\partial X}={\left(I-\rho W\right)}^{-1}\beta =\underset{W^{1}orI}{\underbrace{I\beta }}+\underset{W^{2}}{\underbrace{\rho W\beta }}+\underset{W^{3}}{\underbrace{\rho^{2}W^{2}\beta }}+\ldots +\underset{W^{N}}{\underbrace{\rho^{N}W^{N}\beta }}$$

where ***I***, or **W**^1^, a *n* × *1* vector of ones^7^, *β* is a vector of coefficients, and **W**^2^, **W**^3^,…, **W**^N^ represent the higher order neighbours, from the closest to the furthest.

LeSage and Pace (2009) define the average direct effect as the average of the diagonal elements of (8), and the average indirect effect as the average of the off-diagonal elements. The sum of the average direct and indirect effects gives the average total effect.

In the spatial lag model, the key parameter through which global spillovers arise is the spatial autoregressive coefficient *ρ* (with |*ρ*| < 1). This is reflected in the spatial multiplier ${\left(I-\rho W\right)}^{-1}$, which traces the effect of the linkages between the land consumption levels of neighbouring cantons. The spatial multiplier, as shown in equation (8), can also be expanded to determine the impacts that the explanatory variables themselves have over the higher orders of contiguity (Jensen and Lacombe, 2012). The powers of the autoregressive parameter, *ρ*, in fact, ensure that the marginal effects of a variable decreases with higher orders of contiguity, hence satisfying the second condition of the Tobler’s (1970) First Law of Geography: near things are more related than distant things. This partitioning effect provides spatial econometrics implications in respect to the impacts arising from the changing explanatory variables on different orders of neighbours.

The following empirical study not only concentrates on the investigation of direct, indirect (spillover) and total effects, but also carries out a partitioning analysis for orders of neighbours.

## 4 Empirical results

The first step of the empirical analysis, as shown in the previous paragraph, is the selection of the model and spatial weight matrix. Table 1 shows the Bayesian marginal probability estimation that allows us to select both. The Bayesian marginal probability sums to 1 as in Elhorst and Ensar (2017), and the selection is based on the highest value. The results show that models estimated through a spatial weight matrix based on a decay function always perform better than k-neighbours, confirming the intuition that nearer areas have a higher weight than more distant ones, which is in line with Tobler’s (1970) First Law of Geography. The choice of the cutoff, based on the last column of Table 2, is established at 100 km. The model chosen, as anticipated in the previous paragraph, is clearly a spatial lag, because it has the highest Bayesian marginal probability independently from the **W** matrix.

**Table 1:** Gini and Moran’s I of the share of residential permits over population.

	2007	2008	2009	2010	2011	2012	2013	2014	2015
Gini	0.9091	0.8863	0.8876	0.8563	0.9325	0.9217	0.9304	0.9262	0.9263
Moran's I	0.056***	0.169***	0.061***	0.129***	0.038**	0.101***	0.0470***	0.0266**	0.0366**

Note: *p ≤ 0.05; **p ≤ 0.01; ***p ≤ 0.001. Moran’s I based on 1000 randomizations.

**Table 2:** Bayesian marginal probability estimation for model and **W** selection.

Cont. matrix	cutoff	SLX	Sp. lag	Sp. Durbin	Sp. Error	Sp. Durbin Err.	Sum
k-nearneigh	k = 3	0	0.014592	0	4.25E-27	0	0.014592
	k = 4	0	0.015128	0	6.56E-24	0	0.015128
	k = 5	0	0.016048	0	1.41E-21	0	0.016048
	k = 6	0	0.015183	0	7.67E-21	0	0.015183
	k = 7	0	0.015250	0	4.57E-20	0	0.015250
	k = 8	0	0.017471	0	1.63E-18	0	0.017471
	k = 9	0	0.017237	0	3.17E-18	0	0.017237
	k = 10	0	0.015473	0	6.41E-19	0	0.015473
	k = 11	0	0.015229	0	1.30E-18	0	0.015229
	k = 12	0	0.014864	0	1.62E-18	0	0.014864
	k = 13	0	0.014559	0	2.15E-18	0	0.014559
	k = 14	0	0.014078	0	1.58E-18	0	0.014078
	k = 15	0	0.013591	0	1.56E-18	0	0.013591

Gaussian decay function	10 km	0	0.018084	0	2.58E-39	0	0.018084
	20 km	0	0.016913	0	1.73E-39	0	0.016913
	30 km	0	0.019507	0	8.38E-31	0	0.019507
	40 km	0	0.018523	0	2.54E-27	0	0.018523
	50 km	0	0.023733	0	2.44E-22	0	0.023733
	60 km	0	0.023242	0	4.00E-21	0	0.023242
	70 km	0	0.021571	0	3.19E-19	0	0.021571
	80 km	0	0.020796	0	1.55E-18	0	0.020796
	90 km	0	0.023866	0	1.17E-17	0	0.023866
	*100* km	*0*	*0.023950*	*0*	*4.15E-17*	*0*	*0.023950*
	110 km	0	0.021753	0	1.10E-16	0	0.021753
	120 km	0	0.021712	0	1.92E-16	0	0.021712
	130 km	0	0.021221	0	2.73E-16	0	0.021221
	140 km	0	0.020401	0	3.18E-16	0	0.020401
	150 km	0	0.018844	0	1.90E-16	0	0.018844

Bisquare decay function	10 km	0	0.016826	0	2.12E-39	0	0.016826
	20 km	0	0.015020	0	1.33E-39	0	0.015020
	30 km	0	0.014233	0	9.89E-40	0	0.014233
	40 km	0	0.018748	0	8.79E-39	0	0.018748
	50 km	0	0.019319	0	1.33E-38	0	0.019319
	60 km	0	0.020749	0	4.44E-38	0	0.020749
	70 km	0	0.020430	0	9.13E-38	0	0.020430
	80 km	0	0.021103	0	2.22E-36	0	0.021103
	90 km	0	0.021465	0	1.78E-35	0	0.021465
	100 km	0	0.022249	0	4.30E-33	0	0.022249
	110 km	0	0.023551	0	1.02E-17	0	0.023551
	120 km	0	0.023285	0	2.86E-17	0	0.023285
	130 km	0	0.022963	0	5.71E-17	0	0.022963
	140 km	0	0.021730	0	9.65E-17	0	0.021730
	150 km	0	0.021222	0	1.38E-16	0	0.021222

Weighted k-nearest neighbours	k = 3	0	0.014055	0	2.85E-40	0	0.014055
	k = 4	0	0.014181	0	2.85E-40	0	0.014181
	k = 5	0	0.014052	0	2.85E-40	0	0.014052
	k = 6	0	0.014319	0	2.85E-40	0	0.014319
	k = 7	0	0.014242	0	2.85E-40	0	0.014242
	k = 8	0	0.014243	0	2.85E-40	0	0.014243
	k = 9	0	0.014307	0	2.85E-40	0	0.014307
	k = 10	0	0.014140	0	2.85E-40	0	0.014140
	k = 11	0	0.014298	0	2.85E-40	0	0.014298
	k = 12	0	0.014066	0	2.85E-40	0	0.014066
	k = 13	0	0.014009	0	2.85E-40	0	0.014009
	k = 14	0	0.014205	0	2.85E-40	0	0.014205
	k = 15	0	0.014171	0	2.85E-40	0	0.014171

Sum		0	1.000000	0	1.48E-15	0	1.000000

In Table 3 we report, as a benchmark, the standard Kuznets model that includes only the non-linear GVA per capita. The regressions are estimated through the standard OLS regression and the Spatial Lag Model (column 2 and 3, respectively). The direct, indirect and total effects based on the Spatial Lag Model are in columns 4, 5 and 6. The choice of a fixed effect model is confirmed in both cases by the Hausman test. The randomized Moran’s I (based on 1000 permutations) is highly significant for the OLS model, while it is not for the Spatial lag, indicating that spatial autocorrelation in the residuals is absent in the second model. Furthermore, the Akaike Information Criterion (AIC) shows that there is an improvement in fit when spatial autocorrelation in the dependent variable is modelled properly.

**Table 3:** Regression results of the impact of GVA per capita on building permissions over population.

	OLS		Spatial lag		Direct effects		Indirect effects		Total effects	
log(GVA/pop)	−4.1177	***	−3.2732	**	−3.2732	**	−1.5784	**	−4.8736	**
	(1.4613)		(1.4012)		(-2.3858)		(-2.0305)		(-2.3331)	
log(GVA/pop)^2^	0.2821	***	0.2334	***	0.2350	***	0.1125	**	0.3475	***
	(0.0880)		(0.0840)		(2.8465)		(2.3203)		(2.7664)	
Spatial lag (*ρ*)			0.3283	***						
			(0.0503)							
Spatial multiplier			0.4888							
Flex point GVA/pop (USD)	1478		1110							
Time dummies	yes		yes							
Cantonal dummies	yes		yes							
Observations	1989		1989							
AIC	7274.7		7237.6							
Hausman test (p-value)	12.414 (<0.001)		19.210 (<0.001)							
Moran test (p-value)	0.074 (<0.001)		−0.007 (0.799)							
Breusch Pagan test (p-value)	3.9934 (0.0457)		3.6523 (0.0560)							

Note: *p ≤ 0.10; **p ≤ 0.05; ***p ≤ 0.01. Spatial multiplier is calculated as [(I-*ρ***W**)^-1^]-1. Std. errors in parenthesis for OLS and Spatial lag model (columns 2 and 3). Z-values based on 1000 permutations in parenthesis for the direct, indirect and total effects (columns 4, 5 and 6).

In both cases, there is evidence of nonlinear behaviour of GVA per capita with respect to land use. Contrary to expectations, the curvature is convex, and not concave, which stands for higher levels of land consumption for higher levels of wealth.^8^ This means that the turning point after which higher wealth implies less land consumption will never be reached. These estimates, although contrast to some previous literature (see, among others, Skonhoft and Solem, 2001, Culas, 2007, Liu and Guo, 2015), are in line with those found by Bimonte and Stabile, 2017a, Bimonte and Stabile, 2017b. Anyway, to check the consistency of these results, in case they arise, for example, from some kind of misspecification, we include two additional regressions, as shown in Eq. (1), i.e. population density and the share of GVA in market sector, that are likely to be correlated to land use.

Table 4 is structured like Table 3. The choice of a fixed effect model is also confirmed in this case by the Hausman test, and the randomized Moran test is not significant for the spatial lag model. Furthermore, the Akaike Information Criterion (AIC) shows an improvement with respect to the models estimated in Table 3, and the Breusch-Pagan test is close to 10% in the spatial lag model, pointing to a very weak heteroscedasticity in the residuals. These test statistics lead us to choose the model that includes the additional explanatory variable as the best one, and it is on this that the discussion in the following the paragraph is based.

**Table 4:** Regression results of the impact of GVA per capita, population density and share of market sectors on building permissions over population.

	OLS		Spatial lag		Direct effects		Indirect effects		Total effects	
log(GVA/pop)	−4.0106	***	−3.4045	**	−3.4331	**	−1.6064	**	−5.0295	**
	(1.4658)		(1.4045)		(-2.5043)		(-2.4784)		(-2.4230)	
log(GVA/pop)^2^	0.2752	***	0.2395	***	0.2410	***	0.1128	**	0.3538	***
	(0.0883)		(0.0840)		(2.9098)		(2.8800)		(2.7962)	
log(pop dens)	0.0556		0.0517		0.0520		0.02434		0.0764	
	(0.6570)		(0.0610)		(0.8794)		(0.8269)		(0.8681)	
share market sect	1.6869	**	1.5960	**	1.6064	**	0.7516	*	2.3580	**
	(0.6954)		(0.7091)		(2.2779)		(1.8702)		(2.1527)	
Spatial lag (*ρ*)			0.3232	***						
			(0.0503)							
Spatial multiplier			0.47754							
Flex point GVA/pop (USD)	1460		1221							
Time dummies	yes		yes							
Cantonal dummies	yes		yes							
Observations	1989		1989							
AIC	7271.6		7235.8							
Hausman test (p-value)	39.189 (<0.001)		42.202 (<0.001)							
Moran test (p-value)	0.061 (<0.001)		−0.008 (0.867)							
Breusch-Pagan test (p-value)	7.1786 (0.0664)		6.314 (0.0964)							

Note: *p ≤ 0.10; **p ≤ 0.05; ***p ≤ 0.01. Spatial multiplier is calculated as [(I-*ρ***W**)^-1^]-1. Std. errors in parenthesis for OLS and Spatial lag model (columns 2 and 3). Z-values based on 1000 permutations in parenthesis for the direct, indirect and total effects (columns 4, 5 and 6).

**Fig. 2:**
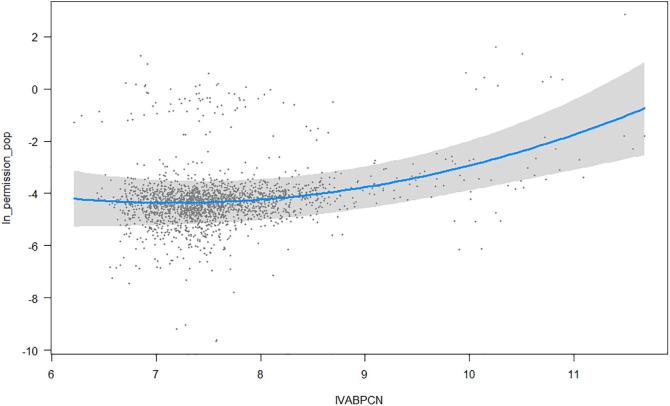
Marginal effect of GVA per capita over the building permissions over population. Shaded area represents the confidence interval.

The convex relationship shown in Fig. 2 between construction permits and economic development is confirmed in Table 4 and, regarding the additional variables, we have, as expected, a positive relationship with the productive sector, while population density has no effect. The latter can be explained by the fact that urbanization has no link with the overcrowding phenomena because people start to construct in the suburbs within and outside the canton, generating a sprawling effect (see, for example, Parés-Ramos et al., 2013).

The size of estimated parameters is upward biased in the OLS model because the spatial lag *ρ* is not accounted for (Mobley et al., 2009), leading to an incorrect turning point equal to 1460 USD instead of 1220 USD. In the first case, the first 35% of poorer cantons would be on the left-side of the turning point, i.e. their land consumption decreases as they become richer. The spatial lag model, instead, shows that only the poorest 25% of cantons are on the left-side of the turning point. Put into another perspective, according to the OLS estimates there would be 14.5% fewer cantons that are experiencing extensive land use.

The spatial lag model allows us to estimate the average direct, indirect and total effects due to the spatial multiplier. Furthermore, we can estimate the pseudo p-values through randomization. The values of the direct effects are very close to those of the coefficients, while the total effects are larger because they account also for the indirect effects, which are equal to the estimated coefficient multiplied by the spatial multiplier equal to 47.75%. These results mean that land use in a certain canton is a function not only of the stage of development of that canton, but also of the stage of development of the surrounding cantons. This phenomenon, the so-called spatial spillover, can be explained with the fact that an increase of income in these neighbourhoods might cause people to decide to invest not only where they live, but also in the neighbouring cantons, raising the number of building permits in both places. Analogously, a shock that rises the available income in one canton has repercussions in the real estate market of the neighbouring cantons, and back to the first one through the spatial multiplier.

The analysis of the direct and indirect effect performed in the previous part of this paragraph does not, however, tell us how important the neighbours are for land use. The partitioning techniques by LeSage and Pace (2009) allow us to calculate the coefficient estimates by different neighbouring orders in Table 5.

**Table 5:** Spatial partitioning results of direct, indirect and total effects of GVA per capita, population density and share of market sectors on building permissions over population.

	Direct							
	log(GVA/pop)		log(GVA/pop)^2^		log(pop dens)		share market sect	
W^1^	−3.41096	**	0.23946	***	0.05169		1.56900	**
	(-2.47827)		(2.87961)		(0.87453)		(2.20141)	
W^2^	0.00000		0.00000		0.00000		0.00000	
W^3^	−0.01760	*	0.00124	**	0.00027		0.00824	*
	(-1.85297)		(2.02505)		(0.79726)		(1.70538)	
W^4^	−0.00300		0.00021		0.00004		0.00140	
	(-1.49021)		(1.58777)		(0.72753)		(1.39977)	
W^5^	−0.00119		0.00008		0.00002		0.00055	
	(-1.20800)		(1.26840)		(0.65222)		(1.14856)	

	Indirect							
	log(GVA/pop)		log(GVA/pop)^2^		log(pop dens)		share market sect	

W^1^	0.00000		0.00000		0.00000		0.00000	
W^2^	−1.10228	**	0.07738	***	0.01670		0.51576	**
	(-2.26159)		(2.56361)		(0.85098)		(2.02679)	
W^3^	−0.33861	*	0.02377	**	0.00510		0.15844	*
	(-1.85396)		(2.02505)		(0.79725)		(1.70538)	
W^4^	−0.11211		0.00787		0.00170		0.05246	
	(-1.49021)		(1.58777)		(0.72753)		(1.39977)	
W^5^	−0.03601		0.00253		0.00545		0.01685	
	(-1.20800)		(1.26840)		(0.65222)		(1.14856)	

	Total							
	log(GVA/pop)		log(GVA/pop)^2^		log(pop dens)		share market sect	

W^1^	−3.41096	**	0.23946	***	0.05169		1.59600	**
	(-2.47827)		(2.87961)		(0.87453)		(2.20141)	
W^2^	−1.10228	**	0.07738	***	0.01670		0.51576	**
	(-2.26159)		(2.56361)		(0.85098)		(2.02679)	
W^3^	−0.35621	*	0.02501	**	0.00540		0.16667	*
	(-1.85396)		(2.02505)		(0.79725)		(1.70538)	
W^4^	−0.11511		0.00808		0.00174		0.05386	
	(-1.49021)		(1.58777)		(0.72753)		(1.39977)	
W^5^	−0.03719		0.00261		0.00056		0.01741	
	(-1.20800)		(1.26840)		(0.65222)		(1.14856)	

Note: *p ≤ 0.05; **p ≤ 0.01; ***p ≤ 0.001. Z-values based on 1000 permutations in parenthesis for the direct, indirect and total effects in parenthesis.

The partitioning direct impacts show that the role of spatial feedback goes beyond the zero-order neighbouring, which contributes to the direct impacts (the diagonal elements in the spatial weight matrix). Although significant, the feedback effect of the immediate neighbours^9^ (**W**^3^) accounts for <1% of the change,^10^ which means that, for direct impacts, only the immediate neighbours play role, and even this is very limited.

With respect to the indirect impacts, the estimates are significant only for the second and third order and notably decrease in size. This demonstrates that a shock has a limited extent over space, a conceivable circumstance in a context in which, as shown, land consumption is particularly concentrated and jeopardised (see Fig. 1).^11^ This also generates a small feedback effect.

As a robustness check, in Table A1 in Appendix A, we estimate a GMM model using sequential moment conditions where lagged levels of the variables are instruments for the endogenous differences and the parameters (see Arellano and Bond, 1991). To choose between GMM-Diff (Arellano and Bond, 1991) and system GMM (Blundell and Bond, 1998) we use the Difference-in Hansen test (Hansen-diff). This test checks the validity of a subset of instruments and is used by Bouayad-Agha and Védrine (2010) to verify if additional instruments used in the system GMM estimation improve that of the GMM-Diff. The test, based on a chi-squared distribution, rejects the null hypothesis, pointing for a GMM-Diff model. The results are in line with the previous estimations in terms of significance, parameter sign and size.

## 5 Discussion and conclusions

The study analyses the nonlinear relationship between land consumption and economic development for 221 cantons in Ecuador using a spatial econometric approach with panel data. Compared with previous studies, we use detailed territorial data, and we exploit their spatial features using ad hoc econometric tools, identifying with a high degree of precision the appropriate spatial weight matrix and spatial model. This, in turn, allows us to identify the magnitude and type of the spatial spillovers, thus generating useful information for setting suitable territorial policies.

The Ecuadorian case study is of particular interest because it shares some characteristics of other Latin American countries while also having some peculiarities related to the implementation of a legislative framework that puts a particular emphasis on the environment.

The results do not support the inverted U-shaped hypothesis of Kuznets curve for land use. In contrast, the curvature is convex, which means higher levels of land consumption for higher levels of wealth, and no turning point where land consumption starts decreasing as wealth increases.

Our finding, i.e. an inverted EKC, is consistent with the findings of Bimonte and Stabile, 2017a, Bimonte and Stabile, 2017b for Italy, and extend them accounting for spatial spillovers, whose strength, coherently with literature (Brueckner, 1998), is limited in space and equal, in our specific case, to 100 km. This means that land use in a canton is correlated to the land use of the closest neighbours. In particular, an improvement in GVA per person has a more than proportional impact in both the cantons where this improvement happens and in the closest ones, inversely proportional to the distance (and no further than 100 km).

A set of factors may contribute to explain excessive land use and spillovers causing the suburbanization and urban fragmentation occurring in Ecuador (see Alova and Burgess, 2017), and the related environmental problems.

From one side, Pendall (1999) and Carruthers and Ulfarsson (2002) find a relationship between decentralized or fragmented governance settings, i.e. political fragmentation, and sprawl. Kim and Hewings (2013) confirm that political fragmentation leads to a higher land-use conversion, and Bluestone (2008) that it prevents the region’s ability to reduce ozone levels.

In spatial context, indeed, local government might find it convenient to compete by granting additional building permits so as not to leave all the advantages of additional revenues to the neighbour governments. Alternatively, they can imitate neighbours' policies as found, for example, by Revelli (2001) that finds large and significant spatial interactions among districts in Great Britain for property tax rate and, more recently by Muñoz (2016) for Colombian municipalities. Regarding the Ecuadorian case, the country is characterized by a particularly decentralized local and regional governance^12^ and by a weak institutional framework; it ranks 117th among 137 countries for transparency of government policymaking (Schwab and Sala-i-Martín, 2017: 109), and 114th among 180 countries for corruption (Transparency International, 2018: 3) The lack of institutional strength can contribute to political fragmentation, lack of horizontal and vertical institutional coordination and, definitively, to a bad management of land by local administrations that can lead to self-construction and “urbanistic freedom” related to builders’ noncompliance with planning regulations (Dobbs et al., 2019).

Another channel that can lead to excessive land consumption is related to the control on residential development that increases price of new housing, rising housing supply into adjacent places where growth controls are not imposed, and prices are lower (Levine 1999). In Ecuador, the accelerated growth of the urban population, which rose from 55% to 64% between 1990 and 2014 (United Nations, 2014), migration from the countryside to the cities (Alvarado et al., 2017), the general improvement of the economic conditions and in-migration^13^ caused an increase in demand for housing and for real-estate investment, producing speculation in the real-estate market, especially in the most dynamic and developed urban centres (Angel, 2008, Jokisch, 2002). This favoured high-income households at the expense of low-income ones, which were relegated in the suburbs.^14^ The latter experienced difficulties in accessing land for, at least, three reasons. The first is the increase of its value (Blanco et al., 2014). The second is because “local elites or wealthier neighbours often exert influence on local governments to enact regulations that restrict land and housing supply for low-income households” (Bouillon, 2012: 148). The third, finally, is that the norms governing minimum lot size and infrastructure requirements can preclude lower-income groups from competing for space through densification (Jaramillo, 1999).

An additional source that can lead to an excessive land use and spatial spillovers is the automobile diffusion, automobile fuel subsidies (Su and DeSalvo, 2008), and the improvement of infrastructure (Baum-Snow, 2007, García-López et al., 2015, Ghani et al., 2014, Henderson and Kuncoro, 1996). These factors contribute to decentralization and suburbanization connecting workers in low densely populated areas to nearest denser locations where business activities are located. Alternatively, due to the fall in transport costs, firms can move non-core activities to remote locations, maintaining the access to the central areas. In Ecuador, motorized vehicles grown by 110% from 2008 to 2015 reaching 1,925,368 units, of whom 68% are cars, and 22% motorbikes (INEC, 2014, INEC, 2015). This is probably leaded, in addition to the general improvement of economic conditions, also by the high subsidies to combustibles that, over the period 2011–2013, accounted for around 7% of GDP (Di Bella et al. 2015). Finally, regarding the improvement of infrastructure, the share of public investment rose from 2.2% in 2007 to 7.4% in 2013, falling down to 4.9% in 2015 (Ministry of Finances, 2018), leading to an expansion of paved roads and better connections between urban and rural areas that ranked the country 29th over 137 countries according to Schwab and Sala-i-Martín (2017: 109).

The environmental impacts of excessive land use and related spillovers that lead to the increase of low density urbanized areas, urban sprawl and fragmentation, are at least of two types: a first type is directly related to the phenomenon, and a second type is related to the opportunity costs of the lack of denser urban areas.

The first type of environmental impacts are related to the fact that urban fragmentation, together with ‘leapfrog’ and discontinuous development (Heim, 2001) the development of roads, railways, and other impervious surfaces causes habitat fragmentation (Swenson and Franklin, 2000, Scolozzi and Geneletti, 2012, Li et al., 2010). This causes fragmentation in socio-ecological systems and habitat loss, affecting directly biodiversity and ecological processes (Fahrig, 2003, Laurance et al., 2011, Haddad et al., 2015, Wilson et al., 2016). According to Alberti and Marzluff, 2004, Alberti, 2005, Grimm et al., 2008, fragmentation isolates habitats by destroying crucial corridors, and reduces or eliminates culturally-relevant open spaces and vegetation (de la Barrera and Henríquez, 2017). This is particularly relevant for Latin America, one of the most urbanized and biologically diverse regions in the world Dobbs et al. (2019), and even more for Ecuador, at the top list of world’s biodiversity hotspots for vertebrate species, endemic vertebrates, and endemic plants (Myers et al., 2000). Furthermore, *peri*-urban sprawl increases car use and the related pollution (Grazi et al., 2008) and prevents densification, and firm agglomeration which might reduce the intensity of the industrial CO_2_ emissions (Chen et al., 2018).

Peri-urban sprawl is also a source of limited sanitation and poor housing conditions, increasing also health risks and pollution of rivers and streams (Torres et al., 2007, Satterthwaite, 2003). The environmental risks are also related to the *peri*-urban occupation of environmentally sensitive areas like volcanic areas around cities like Quito and around Ambato.

The opportunity cost is measured as the lack of positive environmental spillovers that would have been generated by agglomeration effects. The spillovers due to a denser urban environment, as shown by Antonioli et al. (2016), are an engine of environmental innovation. The authors point that firms’ geographical agglomeration is a precondition to supporting environmental innovation due to knowledge flows. Zhang et al. (2018) find relevant urban eco-efficiency spillovers in a panel of 105 Chinese cities and a positive effects of technological innovations on urban eco-efficiency, confirming what observed by Ghisetti and Quatraro (2017) who show that technological innovations, especially green technologies, promote green development by reducing pollutant emissions and the production wastes (Blum-Kusterer and Hussain, 2001).

In line with Sustainable Development Goal number 11 (UN, 2015), to revert the trend that makes land use growing more than proportionally of economic development, and generating negative spatial spillovers that lead to urban and environment fragmentation, a set of measures ensuring sustainable urban development and enhancing inclusive and sustainable urbanization should be adopted.

These measures are of two types: the first aimed at limiting land use and its negative environmental spillovers, and the second aimed at promoting positive environmental spillovers.

Regarding the first type of measures, these include strengthening national and regional development planning (SDG 11.a) and implementing integrated policies and plans towards inclusion, resource efficiency, mitigation and adaptation to climate change, resilience to disasters (SDG 11.b). This may be obtained by strengthening the institutional framework and the coordination among neighbours, but also rethinking the main channel through which local governments collect their revenues, lowering the importance of property taxes. Alternatively, the political autonomy of local government might be better regulated to avoid excessive political fragmentation. Another option, aimed at limiting real estate speculation, might be to set-up ad hoc taxes, as the government tried recently to do,^15^ or, to deal with the issue of the lack of good housing conditions, providing incentives for restructuring houses rather than building new ones. The last point, put in a wider context of urban regeneration would improve the living standards in cities and suburbs, mitigating the rise of house pricing. Furthermore, it would reduce urban and the consequent environmental fragmentation.

Another policy option, which should be agreed with the neighbour cantons to avoid freeriding behaviours that lead to negative environmental spillovers, consists in setting an urban area dedicated to a higher density urban development, with the objective of controlling urban sprawl and land use. Alternatively, density regulations, such as lot size zoning and floor area ratio regulation could be implemented. The first, lot size zoning, sets the minimum or the maximum size of the lot in a certain part of a canton. Floor area ratio, instead, fixes the building height or the ratio of the total floor area of the building relative to the aggregate size of the plot on which they are built.

Finally, there is zoning, i.e. the design of areas purely dedicated to business activities, to residential use, and others assigned to mixed land use. Policies on zoning are essential to implement measures to mitigate the proximity of incompatible land uses, to contain urban sprawling via green belts, to curb congestion, and to provide public facilities such as roads, public transport, parking lots, water and sanitation. The cost of the public facilities should be partially covered by private builders, allowing housing prices in sprawling areas to better reflect the social and environmental cost of urban sprawl. Furthermore, to reduce car dependency and its environmental consequences, fuel subsidies should be reduced, on-street parking charges increased reflecting the social cost of parking provision, and public transport and soft mobility infrastructure, such as cycling paths and pavements, should be improved.

An advantage of zoning is that it is able to promote also positive environmental spillovers because firms located in areas dedicated to business activities would enjoy the positive externalities due to proximity to each other, increasing overall productivity and environmental innovation (Antonioli et al., 2016, Hájek and Stejskal, 2018). Environmental innovation, indeed, takes advantage of knowledge spillovers (Ghisetti et al., 2015), and knowledge spillovers spread out easily in presence of firms’ agglomeration (Acs et al., 1994, Anselin et al., 1997). Thus, policy makers could not only take proper push/pull regulatory framework to smooth the “double externality problem” suffered by the environmental innovators,^16^ but also actively promote firms’ agglomeration.

A tentative in the direction of improving land use management and institutional coordination is the creation in 2016 of an autonomous institution at central level called “Superintendence of Territorial Planning, Use and Land Management” that has the task of controlling land use and administration and human settlements carried out by municipalities ensuring a sustainable urban and rural planning. This solution, in light of the cited literature, might work if political decentralization were to be limited, or if independent bodies were to be allowed to monitor bureaucrats’ behaviour. Furthermore, this initiative may be limited in that, if it is not followed by government simplification, i.e. a decrease in the number of government and administrative tiers, it will result in a high risk of uncoordinated rent seeking of bureaucrats, due to the more complex government structures (Fan et al., 2009).

## Figures and Tables

**Table A1:** Diff-GMM regression results of the impact of GVA per capita, population density and share of market sectors on building permissions over population.

	(1)		(2)	
Constant	0.0173		0.0003	***
	(0.0168)		(0.0202)	
log(build. perm. per person)_-1_	0.2060	***	0.1995	***
	(0.0297)		(0.0298)	
log(GVA/pop)	−4.0927	*	−3.9418	*
	(2.2615)		(2.2537)	
log(GVA/pop)^2^	0.2811	**	0.2713	**
	(0.1319)		(0.1314)	
log(pop dens)			0.1986	
			(0.1268)	
share market sect			1.6964	
			(1.4366)	
Spatial lag (*ρ*)	0.3997	***	0.3823	***
	(0.0909)		(0.0914)	
Spatial multiplier	0.6658		0.6189	
Flex point GVA/pop (USD)	1460		1429	
Instruments	32		34	
Observations	1989		1989	
AR (1) test (p-value)	−18.0745 (<0.001)		−17.9530 (<0.001)	
AR (2) test (p-value)	0.5095 (0.6104)		0.3792 (0.7045)	
Sargan test (p-value)	63.3727 (<0.001)		61.0099 (<0.001)	
Wald test	86.9760		91.2950	
(p-value)	(<0.001)		(<0.001)	
Hansen C test (p-value)	110.3773 (<0.001)		109.1761 (<0.001)	

Note: *p ≤ 0.10; **p ≤ 0.05; ***p ≤ 0.01. Spatial multiplier is calculated as [(I-*ρ***W**)^-1^]-1. Robust std. errors in parenthesis.

## Appendix

Table A1.

## Footnotes

1 Ecuador is a country with 16.5 million inhabitants, divided into 24 provinces, 221 cantons or municipalities and 1228 parishes.

2 The Moran’s I varies between the minimum and maximum eigenvector extracted from **W** (roughly −1 and +1). A positive (negative) value points at positive (negative) spatial autocorrelation, i.e. location with similar (dissimilar) values of the variable analysed are located close to each aother. The Moran’s I is based on a spatial weight matrix **W**. Formally, the spatial weights matrix is an *n*×*n* positive matrix, where *n* is the number of regions. In each row *i*, a non-zero element *w_ij_* defines region *j* as being a neighbour of region *i*. By convention, an observation the diagonal elements are zero (*w_ii_* = 0). **W** is based on a Gaussian decay function with cutoff equal to 100 km. The justification of the choice of such a spatial weight matrix is in the following paragraph.

3 The Gini index ranges between 0 and 1, where 0 is perfect equality, and 1 maximum inequality.

4 The OECD definition of market sector includes also agriculture. We do not include the latter because in Ecuador agricultural activity is mainly subsistence in nature.

5 The stability conditions of the Spatial Lag Panel Model are the same as the ones identified for the Spatial Lag Model. In this regard, as far as the spatial weight matrix **W** is concerned, its row and column sums before row-normalization should not diverge to infinity at a rate equal to or faster than the rate of the sample size N (see Kelejian and Prucha, 1998; Elhorst, 2014, p. 11).

6 The feedback effects arise as a result of impacts passing through neighbouring regions and back to the region where the change was originated (from *i* to *j* to *k* and back to *i*).

7 ***I***, or **W**^1^ are the so-called zero-order neighbouring due to the fact that a canton is a first-order (or higher) neighbour to itself (LeSage and Pace, 2009).

8 We checked for the third power of log(GVA/pop), and the related coefficient is not significant.

9 The first term of the series expansion in (6) contains zeros on the off-diagonal elements. For this reason, the direct effects for the zero-order term, i.e. **W**^2^, will always be equal to zero. Similarly, the indirect effects for the “**W**^1^” term, or the neighbours as defined by the spatial weight matrix **W**, are also zero because by definition the spatial weight matrix **W** contains zeros on the main diagonal (Jensen and Lacombe, 2012).

10 For log(GVA/pop), for example, (**W**^3^ for indirect effects)/(∑**W^N^** for indirect effects) = −0.01760/-3.4331=0.005.

11 At this regard, Andrade-Núñez and Aide (2018) highlight that the expansion of the built environment in South America over the period 2001–2011 was around municipalities with less than fifty thousand inhabitants rather than around the largest cities.

12 According to the Art. 1 of the Constitution: “Ecuador is a constitutional State of rights and justice, a social, democratic, sovereign, independent, unitary, intercultural, multinational and secular State. It is organized as a republic and is governed using a decentralized approach […].” Art. 238 of the Constitution adds: “Decentralized autonomous governments shall have political, administrative and financial autonomy and shall be governed by the principles of solidarity, subsidiarity, inter-territorial equity, integration and public participation. Under no circumstances shall the exercise of autonomy allow for secession from the national territory […].”

13 Jokisch (2002: 547) shows that for the case-study of lower Cañar that “the real landscape change of transational migration has been the conversion of much of Azuay and Lower Cañar Provinces into a *peri*-urban landscape of cultivated real estate supporting nonmigrants, children, the elderly and return migrants seeking rest and possibly retirement from hard work in New York”.

14 According to Art. 314 of the Constitution: “The State shall be responsible for the provision of the public services of drinking and irrigation water, sanitation, electricity, telecommunications, roads, seaport and airport facilities, and others as established by law.” Given that by law there is no general obligation for builders to participate to public infrastructure costs when they build new houses, they can be encouraged to behave as freerides. As the costs for public infrastructure are supported by the community, for private builders, if there is demand, it is always convenient to build new houses.

15 The Capital Gains Law tried to prevent land speculation and real estate bubbles by establishing a tax on extraordinary profits. The law has been repealed by popular consultation on February 4, 2018.

16 Ecological innovators face the “double externality problem” whereby they produce positive knowledge spillovers, and in the meantime improve environmental quality reducing negative environmental spillovers (Rennings, 2000; Jaffe et al., 2005). Then, to avoid that firms have less incentives to invest in eco-innovation because they cannot profit the reduced external costs due to the competing environmentally harmful alternatives, governments can correct market failures combining policies that support technology development (supply push) and that create markets (demand pull) for environmental technologies (Norberg-Bohm, 2000).
